# Supplementary material for: Active site specificity profiling datasets of matrix metalloproteinases (MMPs) 1, 2, 3, 7, 8, 9, 12, 13 and 14
Source: Data Brief. 2016 Feb 22;7:299–310. doi: 10.1016/j.dib.2016.02.036 (PMC4777984; doi:10.1016/j.dib.2016.02.036)
Supplement: Supplementary file 10 — Supplementary material [file mmc10.zip › WebPICS_hMMP8_T_1%/P2.html]

 

PICS results


|  |  |
| --- | --- |
| **P2\_D**  28 in 335 sites   8.4 %    effects > 10 perc. pnts.  (vice-versa in brackets)  P2prime\_I: 18.2 (14.5) |  |
| **P2\_E**  29 in 335 sites   8.7 %    effects > 10 perc. pnts.  (vice-versa in brackets)  P3\_P: 24.5 (10.4)   P1\_Q: 14.7 (21.3)   P1prime\_Y: 10.6 (14.0) |  |
| **P2\_F**  23 in 335 sites   6.9 %    effects > 10 perc. pnts.  (vice-versa in brackets)  P2prime\_W: 15.6 (59.8) |  |
| **P2\_L**  51 in 335 sites   15.2 %    effects > 10 perc. pnts.  (vice-versa in brackets)  P3\_A: 10.4 (12.1)   P1\_N: -10.2 (-12.8) |  |
| **P2\_M**  13 in 335 sites   3.9 %    effects > 10 perc. pnts.  (vice-versa in brackets)  P2prime\_M: 16.8 (10.4) |  |
| **P2\_Q**  27 in 335 sites   8.1 %    effects > 10 perc. pnts.  (vice-versa in brackets)  P1prime\_V: 12.6 (10.7) |  |
| **P2\_S**  27 in 335 sites   8.1 %    effects > 10 perc. pnts.  (vice-versa in brackets)  P3\_V: 12.3 (10.1) |  |
| **P2\_W**  7 in 335 sites   2.1 %    effects > 10 perc. pnts.  (vice-versa in brackets)  P2prime\_T: 35.7 (10.4) |  |
| **P2\_Y**  24 in 335 sites   7.2 %    effects > 10 perc. pnts.  (vice-versa in brackets)  P2prime\_Q: 10.7 (12.8) |  |
